# Screening of active components of *Ganoderma lucidum* and decipher its molecular mechanism to improve learning and memory disorders

**DOI:** 10.1042/BSR20232068

**Published:** 2024-07-31

**Authors:** Xiao-tian Zhang, Chun-lei Ji, Yu-juan Fu, Yue Yang, Guang-yu Xu

**Affiliations:** 1 Department of Clinical Laboratory, The Second Hospital of Jilin University, Changchun, Jilin 130000, People’s Republic of China; 2 Specialty in Pharmaceutical Analysis, College of Pharmacy, Beihua University, Jilin, Jilin 132013, People’s Republic of China

**Keywords:** Ganoderma lucidum, Learning and memory impairment, Mechanism of action, Regulatory network

## Abstract

Learning and memory impairment (LMI), a common degenerative central nervous system disease. Recently, more and more studies have shown that *Ganoderma lucidum* (GL) can improve the symptoms of LMI. The active ingredients in GL and their corresponding targets were screened through TCMSP (Traditional Chinese Medicine Systems Pharmacology Database and Analysis Platform) and BATMAN-TCM (Bioinformatics Analysis Tool for Molecular Mechanism of Traditional Chinese Medicine) databases, and the potential LMI targets were searched for through GeneCard (GeneCards Human Gene Database) and DrugBank. Then, we construct a ‘main active ingredient-target’ network and a protein–protein interaction (PPI) network diagram.The GO (Gene Ontology) functional enrichment analysis and KEGG (Kyoto Encyclopedia of Genes and Genomes) pathway annotation analysis were performed on the common targets through DAVID (Database for Annotation Visualization and Integrated Discovery) to clarify the potential molecular mechanism of action of active ingredients in GL. The tumor necrosis factor (TNF) protein was verified by Western blot; Twenty one active ingredients in GL and 142 corresponding targets were screened out, including 59 targets shared with LMI. The 448 biological processes shown by the GO functional annotation results and 55 signal pathways shown by KEGG enrichment analysis were related to the improvement of LMI by GL, among which the correlation of Alzheimer’s disease pathway is the highest, and TNF was the most important protein; TNF can improve LMI. GL can improve LMI mainly by 10 active ingredients in it, and they may play a role by regulating Alzheimer’s disease pathway and TNF protein.

## Introduction

Learning and memory impairment (LMI) is a common degenerative central nervous system disease, and its onset is hidden, its pathogenesis is complex and the number of patients with LMI is increasing year by year, seriously endangering the physical and mental health of the elderly in China [1]. Therefore, its early prevention and treatment are very important. At present, the drugs for the clinical treatment of LMI can not significantly effectively prevent and delay the progress of LMI, and there are some problems in the clinical application of the drugs, such as low therapeutic effect and more adverse reactions [2]. A variety of proteins, such as serine/threonine protein kinases, inflammation- and immune-related proteins, may be potential targets for the treatment of nervous system diseases [3–5]. Traditional Chinese medicine has a significant effect on the symptom relief and course delay of LMI, with the advantages of less adverse reactions and high effectiveness, so the molecular mechanism-related research on the intervention with traditional Chinese medicine in LMI has become increasingly active [6].

*Ganoderma lucidum* (Leyss. ex Fr.) Karst (*Ganoderma lucidum*, GL) is an annual wood-degrading *Basidiomycota* plant [7,8]. GL is rich in multiple active ingredients, including *Ganoderma* polysaccharides, amino acids, polypeptides and triterpenes, of which GL* triterpenes* are widely used in alleviating neurological diseases, anti-inflammation and antioxidation, with significant effects [9–11]. Some studies have shown that other active ingredients in GL can also alleviate the symptoms of LMI [12].

Traditional Chinese medicine is characterized by complex components, multiple pathway targets and synergistic effects. Network pharmacology can explain the therapeutic mechanism of drugs with multiple components and multiple targets from the perspective of genes and signal pathways, and the molecular mechanism of drugs can be built into a visual regulatory network by network pharmacology method, which may be used to more systematically and comprehensively explore the relationship between drugs and diseases by finding the drug targets to guide the research and development of new drugs [13,14].

**Figure 1:**
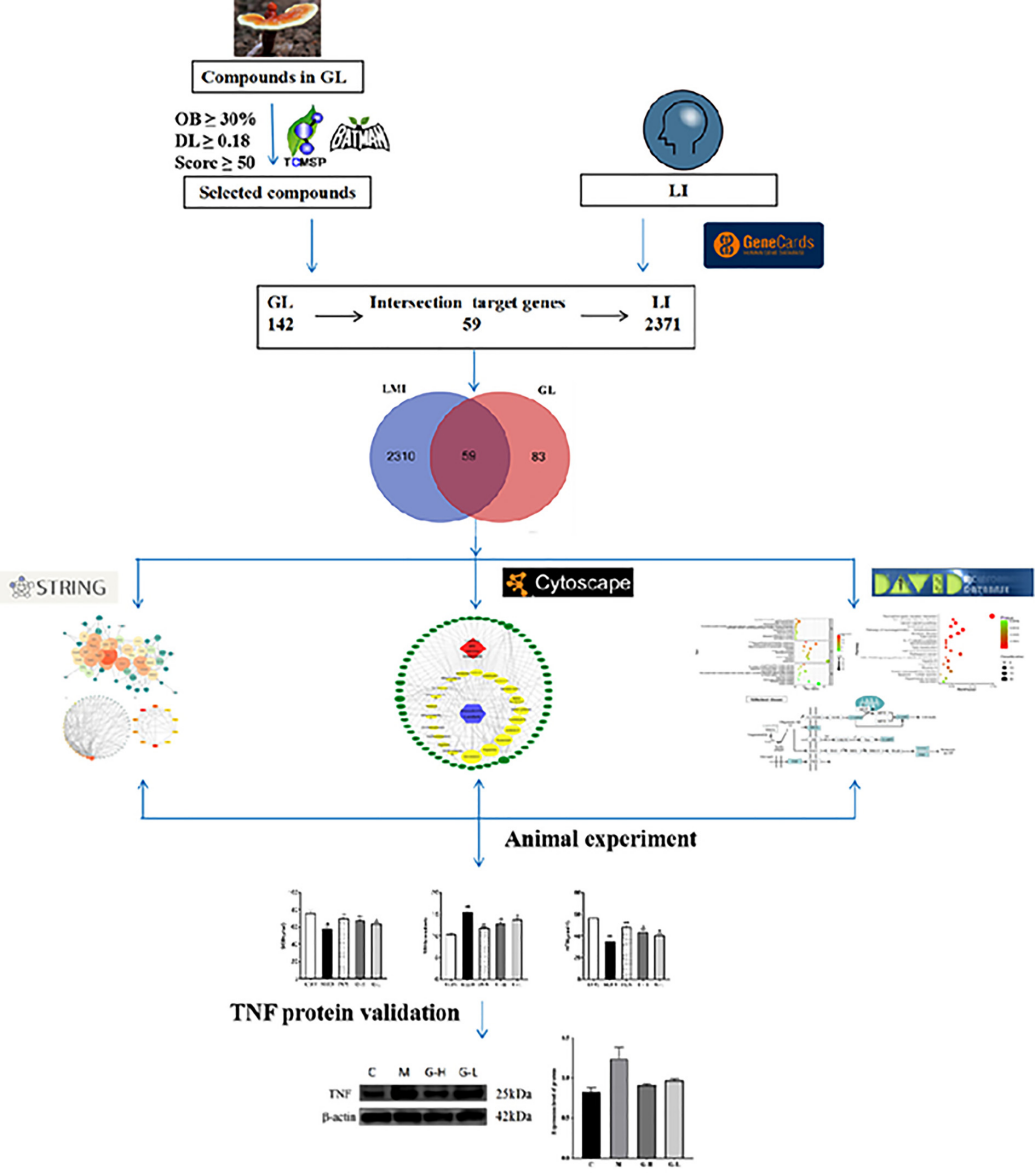
Workflow of target prediction with TNF protein inhibitors in LMI

In the present study, we predicted and analyzed the active ingredients and targets related to LMI in GL, constructed the ‘active ingredient-target’ network and mapped the target protein interaction network, and performed GO (Gene Ontology) analysis and KEGG (Kyoto Encyclopedia of Genes and Genomes) pathway enrichment analysis on the targets, and finally screened the main active ingredients of GL to improve learning and memory impairment and elucidated their potential molecular mechanism of action, which can provide theoretical support for further application of GL. The results showed that the main active ingredients of GL can improve learning and memory impairment and its potential molecular mechanism of action, which can provide theoretical support for the further application of GL. The workflow is shown in Figure 1.

## Methods and materials

### Drugs and reagents

GL (Jilin City Chinese Herbal Medicine Market); Formic acid (Fisher Chemical); SOD, MDA Assay kit (Nanjing Jiancheng Bioengineering Research Institute); ACh Enzyme-linked immunoassay kit (Shanghai Langton Biotechnology Co., LTD); Carbinol (Fisher Chemical); D-gal (Beijing Dingguo Changsheng Biotechnology Co., LTD); Piracetam (Northeast Pharmaceutical Group, Shenyang First Pharmaceutical Co., LTD); QIAzol Cracking buffer (Qiagen,Germany).

### Instrument

Infinite M200 microplate reader (TECAN Group, Switzerland); Desk centrifuge (Eppendorf, U.S.A.); AL204 electronic balance [Mettler-Toledo Instruments (Shanghai) Co., ltd., Shanghai, China].

### Database and software

TCMSP database (https://tcmspw.com/index.php); BATMAN-TCM database (http://bionet.ncpsb.org.cn/batman-tcm/); NCBI database (https://www.ncbi.nlm.nih.gov/); UniProt database (https://www.uniprot.org/); GeneCards database (http://www.genecards.org/); DrugBank database (https://go.drugbank.com/); Cytoscape 3.7.1 software (http://www.cytoscape.org/); DAVID OLAP tool (https://david.ncifcrf.gov/); STRING database (https://string-db.org/); PubChem database (https://pubchem.ncbi.nlm.nih.gov/); PDB database (http://www.pdb.org/); CB-DOCK database (http://clab.labshare.cn/cb-dock/php/download.php).

### Animals

Sixty SPF-grade ICR mice, male, weighing 17–21 g, were divided into four groups for the experiments according to the random grouping method, with 15 mice in each group, namely the blank group (CON), the model group (MOD), and the GL polysaccharide high and low dose administration groups (G-H, G-L). Mice were purchased from Changchun Yis Laboratory Animal Technology Co., Ltd (license number: SCXK-2018-0007), and all experiments were performed according to established animal research guidelines. Experiments were started after 3 days of acclimatization by feeding in separate cages at 20–24°C and 40–60% humidity for 12 h day and night, with free diet and water. These mice were maintained in a temperature- and humidity-controlled environment with a 12-h light–dark cycle and specific pathogen-free conditions. During the experimental period, 300 mg·kg^−1^ D-galactose was injected subcutaneously daily into each group of mice except the blank group [15,16], and an equal volume of saline was injected subcutaneously into the blank group daily; meanwhile, each group of mice was administered daily by gavage, and 300 mg·kg^−1^·d^−1^ Piracetam was given to the positive group and 300 mg·kg^−1^·d^−1^ to the G-H and G-L administration groups, respectively, 100 mg·kg^−1^·d^−1^ of GL polysaccharide, and equal volumes of distilled water were given to the CON and MOD groups.

The mice were put into an euthanasia box (connected to a carbon dioxide cylinder and flow controller), the concentration of input carbon dioxide was 100%, and the flow rate of carbon dioxide was set at 20% of the chamber volume/minute. After the respiratory arrest and eye color fading of the mice, the carbon dioxide was continuously input for five minutes, and then the mice were placed out the euthanasia box for 5 min to ensure the death of mice.

All animal experiments were approved by the ethics committee of Beihua University. All animal experiments took place at Beihua University.

### Preparation of GL aqueous extracts

The GL Sporophore were dried, then cut into cuttings and sieved through four mesh screen, extracted by heating in a water bath at 90°C for 2 h, and centrifuged at 4000 rpm for 15 min. The supernatant was evaporated to 1/4 of the original volume, and centrifuged at 4000 rpm for 15 min under the condition of 4°C with 95% ethanol for 24 h to obtain the crude extract.

### Active ingredients in GL and their target prediction

The active ingredients in GL were screened in TCMSP database and BATMAN-TCM database [17]. The active ingredients with an oral bioavailability (OB) ≥ 30% and a drug-likeness (DL) ≥ 0.18 were screened in TCMSP database, and those with a score ≥ 50 were screened in the BATMAN-TCM database [18]. The conversion of the corresponding gene names of the target proteins corresponding to the obtained active ingredients was performed through NCBI to obtain the gene targets of the active ingredients in GL [19].

### LMI target prediction and key target screening

Using ‘learning and memory impairment’ as the keyword, the known disease targets were screened in Genecards and DrugBank [20]. The duplicate targets were deleted, and mouse homologous genes were searched for in NCBI database.

Active ingredient targets in GL and LMI targets were intersected, and finally the active ingredient and their corresponding LMI targets were found.

### Construction of the regulatory network of ‘active ingredients-LMI targets’

The 59 targets of GL to improve LMI were imported into Cytoscape 3.7.1 software to build a network diagram of ‘active ingredients-LMI targets’ [21]. The node degree values were sorted, and the ingredient-target correspondence was analyzed according to the node degree values of the active ingredients and the targets, respectively.

### GO and KEGG enrichment analysis

In order to study the potential molecular mechanism of GL in the treatment of LMI, the 59 targets of GL to improve LMI were imported into DAVID database, in which *P*<0.05 was set, and the GO function enrichment analysis and KEGG pathway enrichment analysis were performed [22], in which the GO function enrichment analysis mainly included three processes: biological process (BP), cell composition (CC) and molecular function (MF).

### Construction of PPI regulatory network and screening of key genes

The common targets of the 59 targets of GL to improve LMI were imported into STRING database, ‘multiple proteins’ was selected and the species was selected as ‘Mus musculus’ to obtain the data of protein–protein interaction (PPI), and the parameters were imported into Cytoscape software for visual analysis and drawing the PPI network diagram [23]. The centrality of nodes was calculated by using CytoNCA plug-in in Cytoscape software, in which the larger the quantitative value of the parameter, the greater the importance of nodes in the network. Cytohubba plug-in in Cytoscape software was used for the network topology analysis and the calculation of the degree of freedom of the targets.

### Animal sampling and biochemical index testing methods

Mouse eyes were removed using clean forceps, approximately 1 ml of whole blood was taken, centrifuged at 3000 rpm for 15 min at 4°C, serum was separated, stored at −20°C and set aside. Whole mouse brain tissue was collected, weighed and homogenized in a 10% chilled water bath with saline at a ratio of 1:9 (p:v), centrifuged at 3500-rpm for 10 min at 4°C, the supernatant was extracted, stored at −80°C and set aside. The SOD (superoxide dismutase) activity and MDA (malondialdehyde) content in mouse serum were determined according to the kit instructions, and Ach (acetylcholine) in mouse brain tissue was determined according to the ELISA instructions.

### Experimental protocol for TNF protein detection

Take about 50 mg of mouse brain tissue, homogenize, shake, make it completely lysed, centrifuge and collect the total protein solution, refer to the relevant steps of the BCA protein concentration kit to complete the protein concentration determination, make it denatured, make the gel and upload the sample, wait for the concentrated gel to solidify well, perform electrophoresis, wet turn the membrane with activated PVDF membrane, put it into the incubation bath of TBST, shabu-shabu wash, close it at room temperature for 60 min. Add the configured primary antibodies tumor necrosis factor (TNF) (1:1500) and β-actin (1:2000), incubate on a shaker at 4°C overnight, recover the primary antibodies, shabu-shabu the membrane three times with TBST, add TBST again, wash the membrane 15 min × 3 times, add the secondary antibodies diluted (1:5000) to the incubation bath, shake slowly on the shaker, incubate at room temperature for 60 min, wash the membrane with TBSB for 15 min × 3 times min × 3 times, add ECL solution, and develop and fix the film after pressing. The analysis was performed using ImageJ software, and the relative expression of the target protein was expressed by calculating the ratio of the target protein bands to the gray level of the internal reference β-actin.

### Statistical analysis

The data are measured three times, the average value is calculated, and the relative standard deviation (SD) of the three times is obtained. GraphPad Prism 8 software (https://www.graphpad.com/) was used for plotting, *t*-test and variance analysis. *P* < 0.05 was considered statistically significant.

## Results

### Active ingredients in GL and potential targets, and active compounds and potential targets

Through TCMSP database, with OB ≥ 30% and DL ≥ 0.18 as screening conditions, 14 active ingredients in GL and their corresponding 36 targets were screened out, and 6 active components in GL and their corresponding 111 targets were screened out through BATMAN-TCM database. Twenty one active ingredients and 142 corresponding targets were obtained after removing the duplicates and combining the search results.

### GL-LMI target prediction

**Figure 2:**
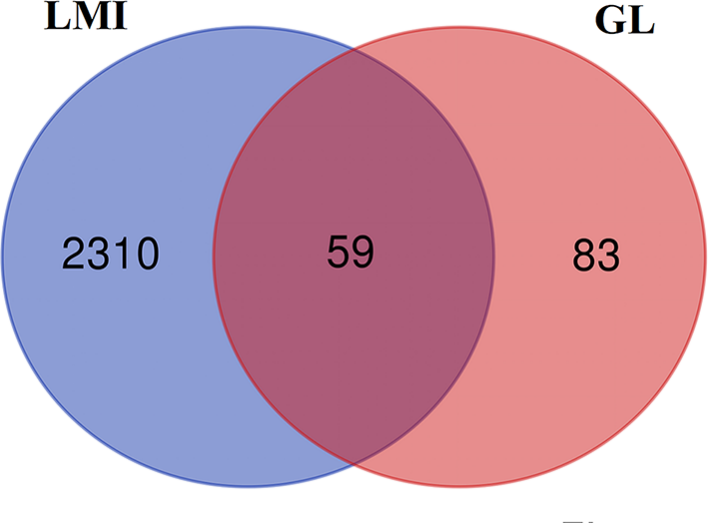
Intersection of GL target genes and LMI genes Blue: LMI targets; Red: targets corresponding to active ingredients in GL.

We collected 2369 LMI-related disease targets from Genecards database. The active ingredient targets and LMI targets were intersected, and finally 59 targets for GL to improve LMI were found (Figure 2). The active ingredients corresponding to the 59 targets were analyzed, and it was found that the targets still corresponded to 21 active ingredients in GL.

### Regulatory network of ‘active ingredients in GL-LMI targets’

**Figure 3:**
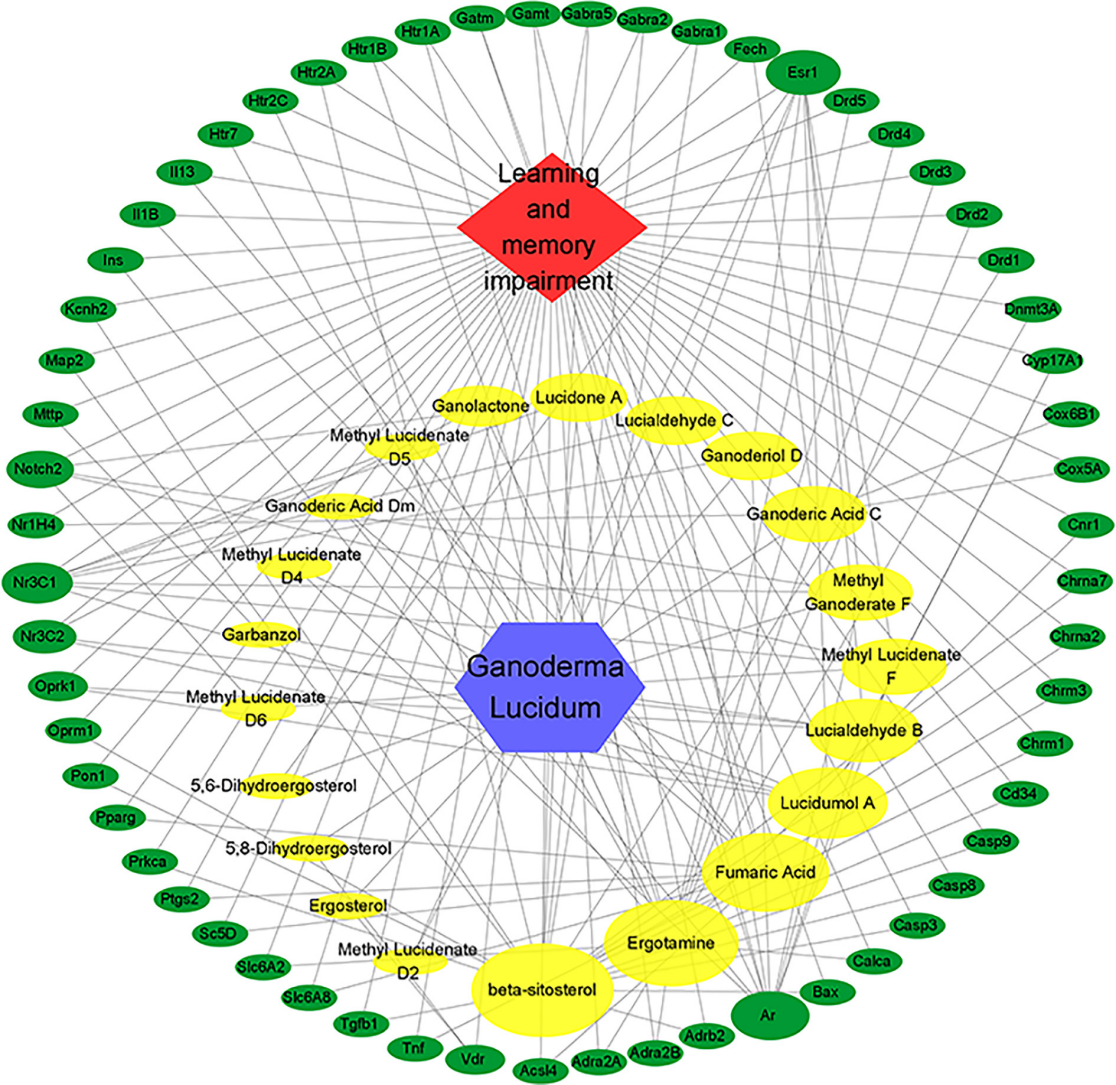
GL-LMI-potential target gene network The size of each node in the network represents the size of its degree value. The gray connecting lines indicate that each node is interconnected.

The regulatory network of ‘active ingredients in GL-LMI targets’, that is, that of the screened 59 LMI targets and their 21 corresponding active ingredients in GL, was constructed by using Cytoscope 3.7.1 software (Figure 3). In the regulatory network of ‘active ingredients in GL-LMI targets’, 82 nodes and 169 edges were found. The ‘degree’ value of a node is defined as the number of edges connected to it in the network, indicating the importance of the node in the network. Therefore, the degree values of all nodes in the network diagram were calculated. The results showed that the top 10 active ingredients mainly included β-sitosterol, ergotamine, fumaric acid, lucidumol A, lucialdehyde B, methyl lucidate F, methyl ganoderate F, ganoderic acid C, ganoderiol D and lucialdehyde C (Table 1).

**Table 1:** Top 10 active ingredients in GL

Number	Compound CID	Molecule ID	Molecule name	OB (%)	DL	Target
1	222284	MOL000358	β-Sitosterol	43.51	0.72	21
2	8223		Ergotamine			17
3	444972	MOL000602	Fumaric acid	17.74	0.01	11
4	475410	MOL011290	Lucidumol A	34.75	0.80	7
5	10343868	MOL011267	Lucialdehyde B	43.12	0.81	6
6	21633085		Methyl Lucidenate F			5
7	21633081	MOL011298	Methyl Ganoderate F	22.71	0.70	5
8	146156325	MOL011186	Ganoderic acid C	22.23	0.79	4
9	15602261	MOL011233	Ganoderiol D	25.46	0.82	4
10	10366713	MOL011268	Lucialdehyde C	42.26	0.81	4

### GO enrichment analysis and KEGG enrichment analysis

**Figure 4:**
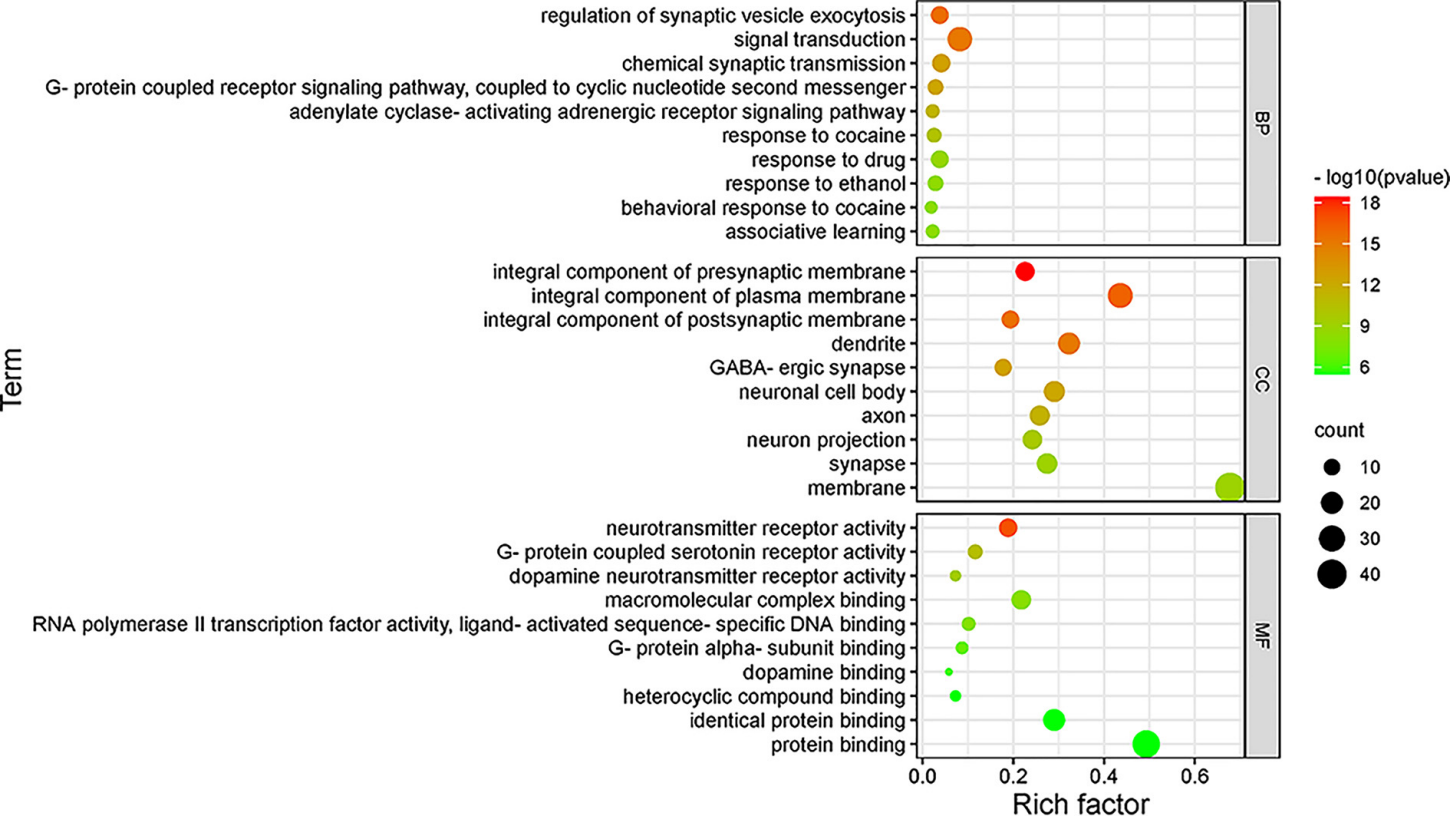
GO enrichment analysis Rich factor refers to the ratio of enriched genes to all target genes, and Term refers to the name of enrichment process.

The GO enrichment analysis on the 59 targets of GL to improve LMI was performed. The results showed that 317 BP related to the improvement of GL on LMI mainly existed in the regulation of synaptic vesicle exocytosis and signal transduction, there were 62 CC, and the targets mainly existed in the integral component of presynaptic membrane and the integral component of plasma membrane, and there were 69 MF, mainly involving neurotransmitter receptor activity and G-protein-coupled serotonin receptor activity. The bubble chart of the top 10 processes in BP, CC and MF was drawn according to the significance of *P*-value (Figure 4).

**Figure 5:**
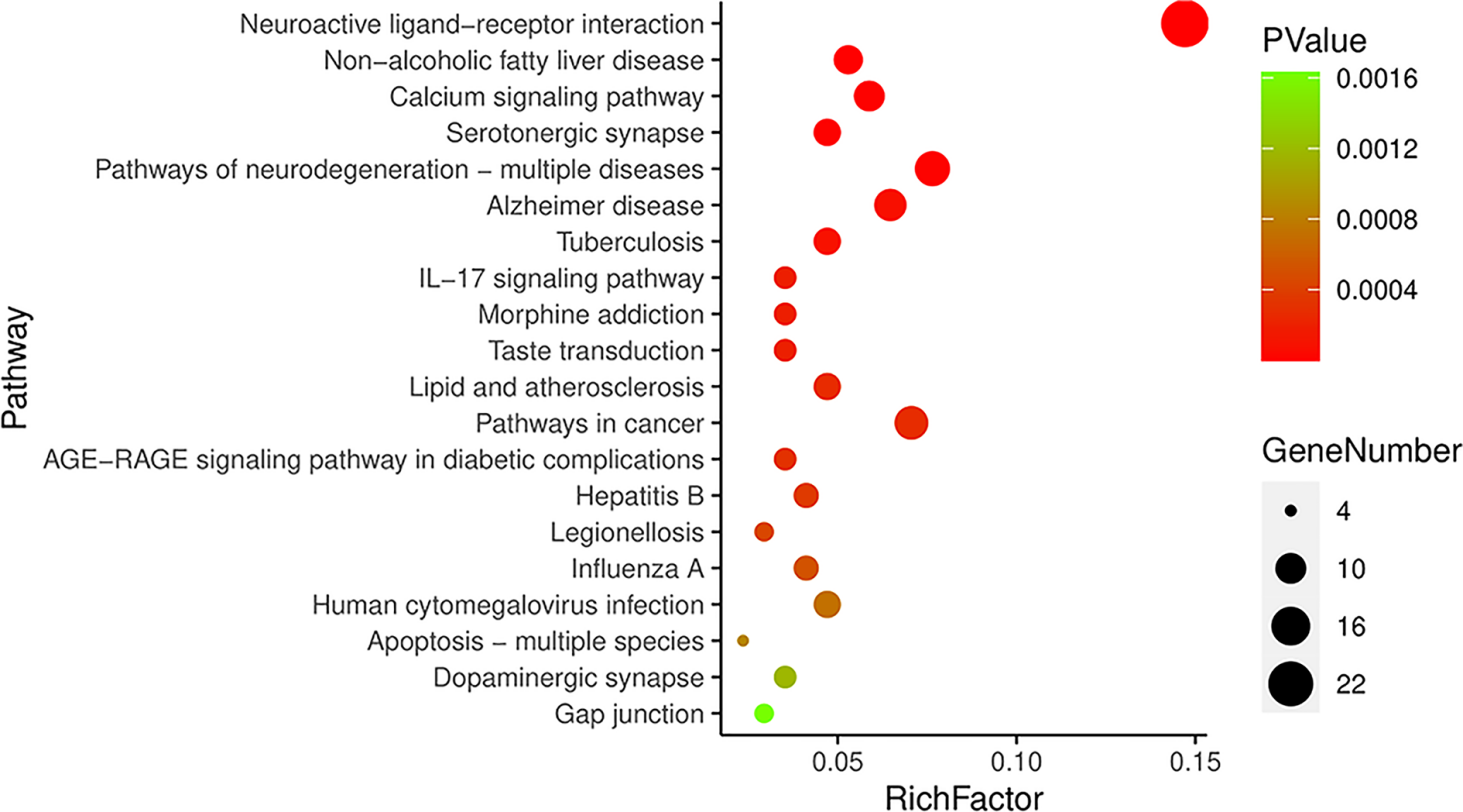
KEGG enrichment analysis on the intersection of active ingredients in GL and LMI genes

**Figure 6:**
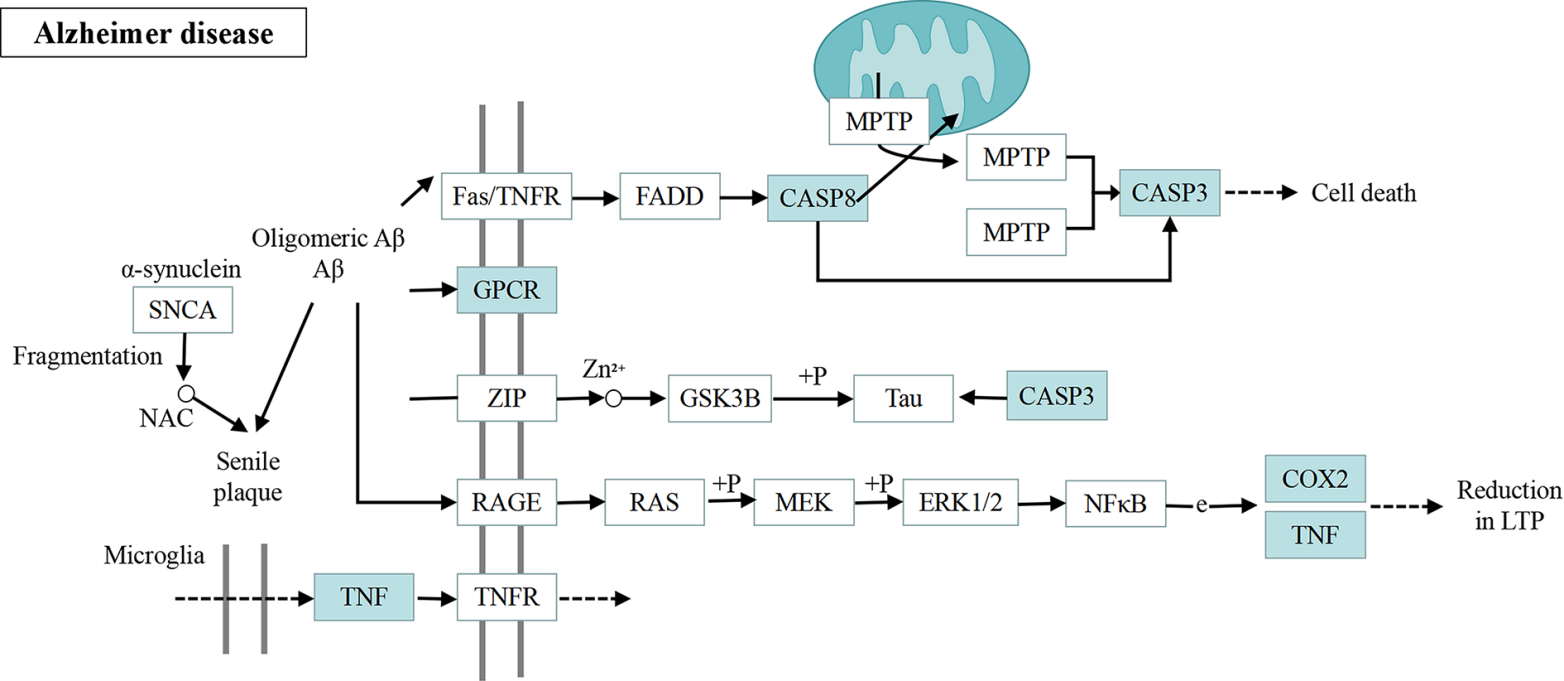
Alzheimer’s disease pathway (only some of the GL improvement LMI related targets are shown) Blue box: 59 screened target genes of GL to improve LMI.

The KEGG enrichment analysis was also performed, and 55 related pathways were found, mainly involving neuroactive ligand–receptor interaction pathway, non-alcoholic fatty liver disease pathway, calcium signaling pathway, and Alzheimer’s disease pathway. The top 20 pathways with the most significant *P*-value were selected for study according to the significance of *P*-value (Figure 5). It was found by references that the relevance of Alzheimer’s disease pathway with learning and memory impairment was highest among these 20 pathways, so it was believed that Alzheimer’s disease pathway might be the regulatory pathway for GL to improve LMI. In addition, it was also found that 5 targets were on this pathway among the previously screened 59 targets for GL to improve LMI, namely, Tnf (TNF), Chrm3 (GPCR), Casp8 (CASP8), Casp3 (CASP3) and Ptgs2 (COX2) (Figure 6).

### PPI regulatory network and screened key genes

**Figure 7:**
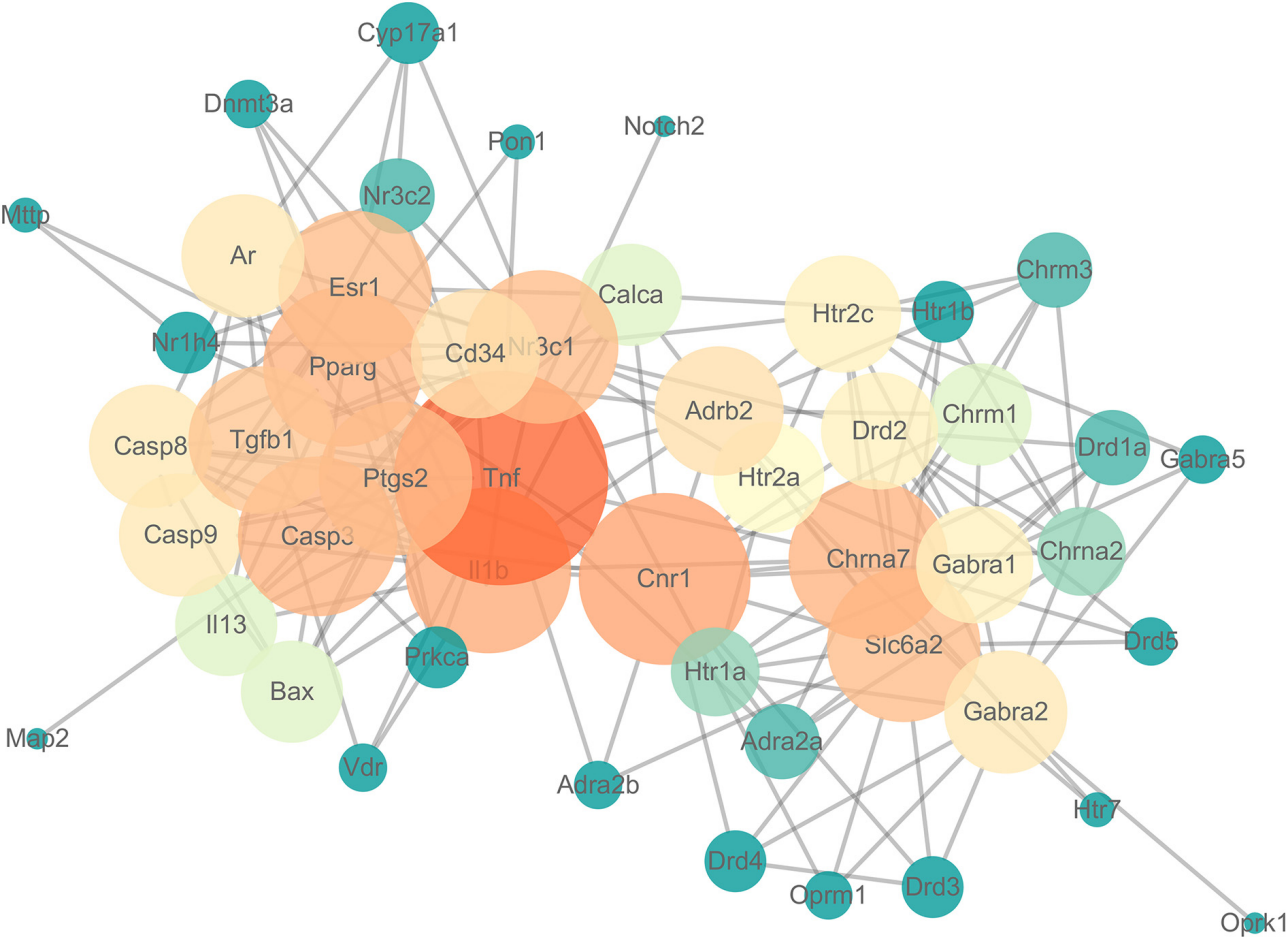
PPI regulatory network

PPI, a protein network composed of individual proteins connected by mutual interaction, can be used to more systematically and comprehensively study the molecular mechanism of diseases and discover new drug targets. In order to understand the PPI, 59 targets of GL to improve LMI were imported into STRING database to obtain the data of PPI network, and the data were visualized with Cytoscape 3.7.1 software to obtain the PPI network diagram (Figure 7).

**Figure 8:**
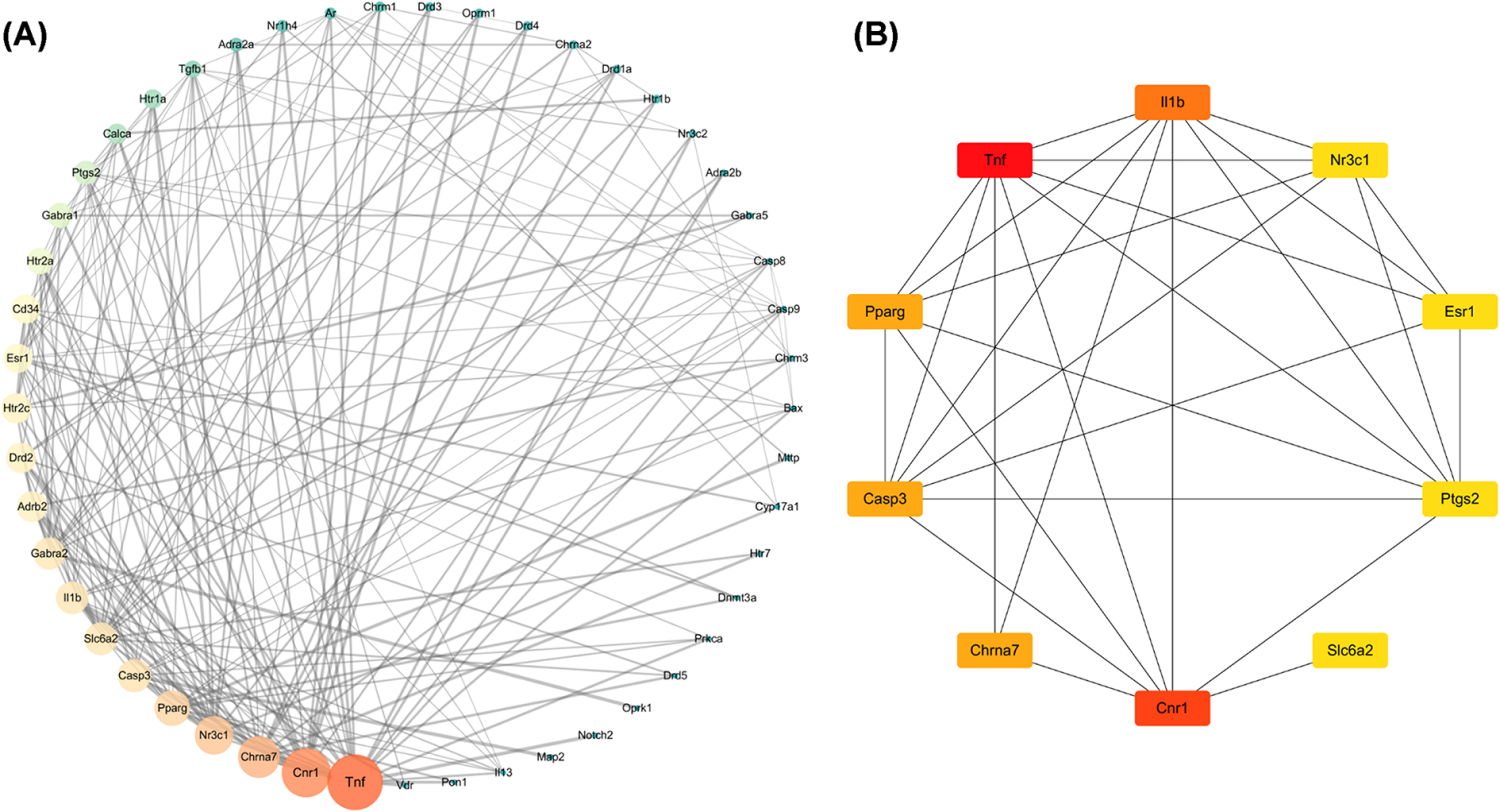
Identification of key sub-networks using Cytoscape (**A**) CytoNca target gene central regulatory network. (**B**) CytoHubba key sub-network.

**Figure 9:**
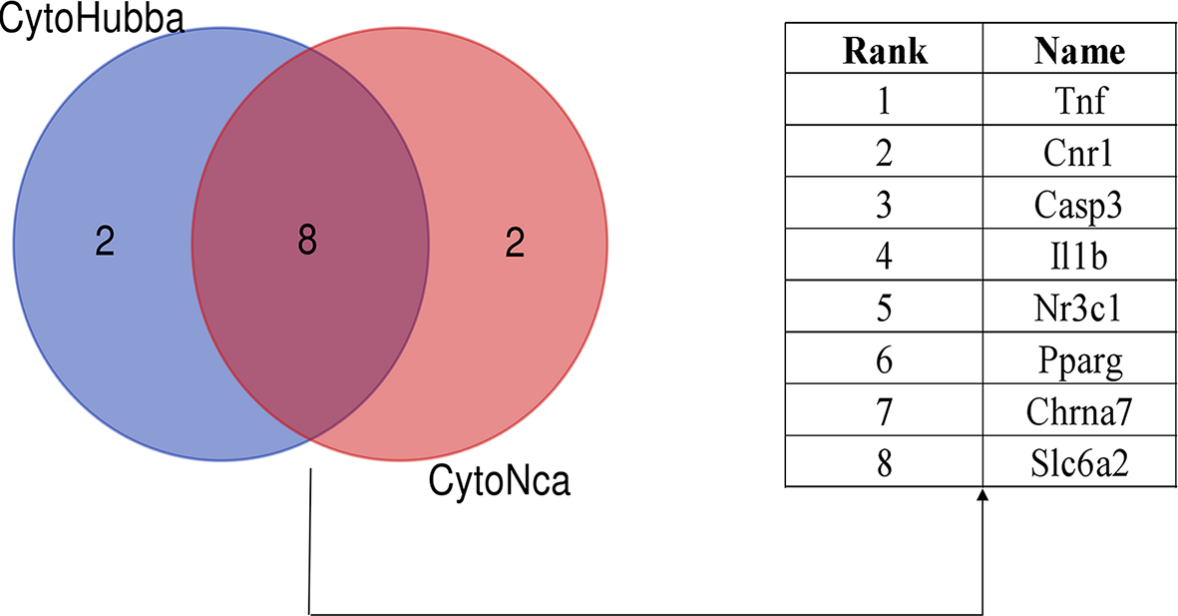
Screening of key genes using Cytoscape plug-in

The PPI network were further analyzed by using CytoNca plug-in in Cytoscape, and the central regulatory network of the target genes was obtained (Figure 8). In addition, the key sub-networks composed of the target genes were obtained using the degree algorithm of CytoHub plug-in in Cytoscape (Figure 9), and eight key genes were obtained by intersecting the results of the two key sub-networks, of which Tnf gene was the most key target (Figure 9).

### Effect of GL on biochemical indices in the brain

Compared with the CON group, the activity of SOD in the serum of mice in the MOD group was significantly decreased (*P*<0.01), while the content of MDA was significantly increased (*P*<0.01); after administration, compared with the MOD group, the activity of SOD in the serum of mice in the POS, G-H and G-L groups was significantly increased (*P*<0.01, *P*<0.05), and the MDA content was significantly decreased (*P*<0.01, *P*<0.05).

**Figure 10:**
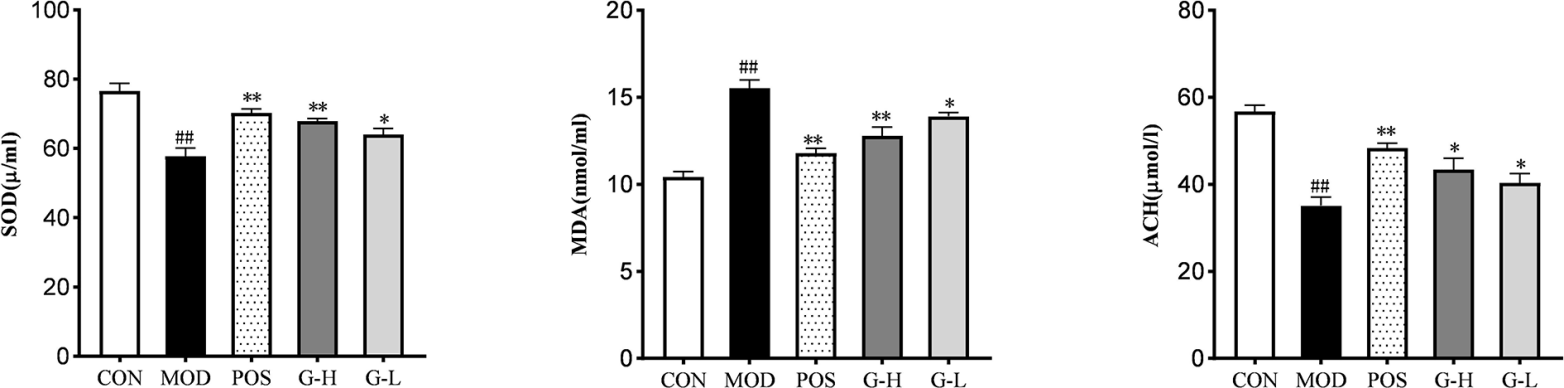
Effects of *Ganoderma lucidum* water extract on SOD, MDA and ACH in brain tissue of D-galactose aged mice Note: Compared with MOD group *P<0.05, **P<0.01; Compared with CON group*^##^P<0.01* (*n*=3, mean ± SD; CON, blank group; MOD, Model group; POS, positive drug group; G-H, Ganoderma lucidum water extract high-dose group; G-L, Ganoderma lucidum water extract low-dose group; SOD, superoxide dismutase; MDA, malondialdehyde; ACH, acetylcholine).

Compared with the CON group, the content of acetylcholine (ACH) in the brain tissue of mice in the MOD group was significantly decreased (*P*<0.01). The content of ACH in the brain tissue of mice in the MOD group increased after administration of the drug (*P*<0.01, *P*<0.05) (Figure 10).

### TNF protein validation

**Figure 11:**
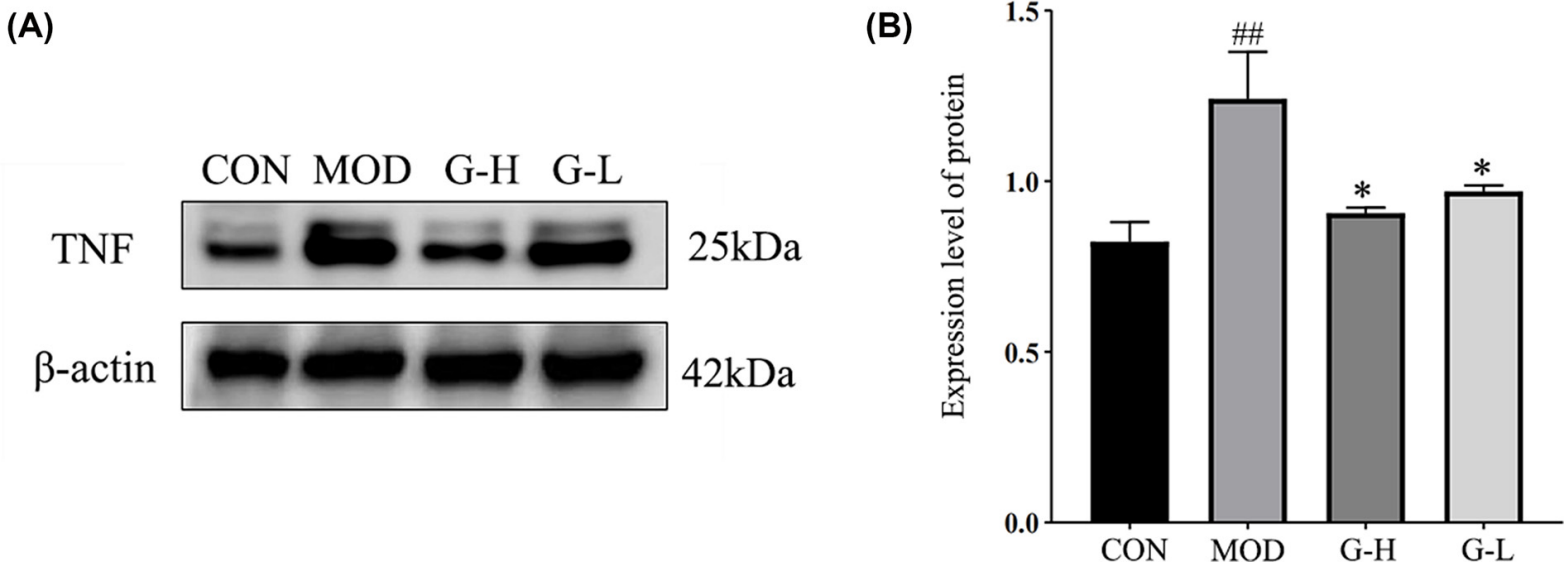
TNF protein expression Left panel: Protein immunoblotting images; right panel: Visualization of bar charts after data analysis; CON, blank group; MOD, Model group; G-H: High-dose Ganoderma lucidum water extract group; G-L: Low-dose Ganoderma lucidum water extract group. Compared with MOD group, ##*P*<0.01; Compared with CON group, *P<0.05 (*n*=3, mean ± SD).

Compared with the blank control group, TNF protein expression was significantly up-regulated in the model control mice (*P*<0.01), compared with the model control group, TNF protein expression was significantly down-regulated in the ganoderma-administered high-dose group and the ganoderma-administered low-dose group (*P*<0.05 or *P*<0.01). (Figure 11)

## Discussion

LMI is a multifactorial neurodegenerative disease involving many cellular and molecular processes [24]. It is one of the most common clinical symptoms of nervous system injury and aging, and has seriously affected the quality of life of patients [25]. In recent years, it has been demonstrated that GL can improve LMI, but there are few studies on its mechanism of action and active ingredients. In the present study, the mechanism of GL in improving LMI was investigated by traditional Chinese medicine network pharmacology methods, and with the help of relevant databases and software.

In the present study, we screened 21 GL active ingredients related to LMI and their corresponding 59 targets to build a ‘Ganoderma lucidum active ingredient-learning memory impairment target’ regulatory network, and found that 10 active ingredients play a major role (β-sitosterol, Ergotamine, fumaric acid, Lucidumol A, Lucialdehyde B, Methyl Lucidenate F, Methyl Ganoderate F, Ganoderic Acid C, Ganoderiol D, Lucialdehyde C). Among them, Lucidumol A, Lucialdehyde B, Methyl Ganoderate F, Ganoderiol D and Lucialdehyde C are triterpenoids all have certain free radical scavenging ability and anti-inflammatory and antioxidant activity [26–31], Therefore, Ganoderma triterpenes may be the key active ingredients for improving LMI. Lucidumol A has good inhibitory activity against aldose reductase (aldose reductase) [32]. Lucialdehyde B and Methyl Lucidenate F have strong cytotoxic properties [33,34]. Ganoderiol D has some free radical scavenging ability and anticancer activity, and some studies have reported that Ganoderiol D can act as an antagonist of MMPs (Matrix metalloproteinases) [35]. Lucialdehyde C belongs to the group of Lanostane triterpenes compounds and has a strong inhibitory effect on α-glucosidase (α-glucosidase) and murine tumor cells [36,37]. Methyl Ganoderate F has some anti-inflammatory activity and may have some effects on PI3K/AKT-Nrf2 pathway [38]. Fumaric acid also has anti-inflammatory effects [39,40], and β-sitosterol, ergotamine and ganoderic acid C all improve neurological related disorders, where the OB and DL of ergotamine are currently unknown and will not be studied in depth here. β-Sitosterol and ganoderic acid C improving learning memory impairment and cognitive impairment in mice [41–43]. β-Sitosterol is a natural phytosterol [44] that can easily penetrate the blood–brain barrier and accumulate in the brain, and its mechanism of action is to improve memory and learning impairment by inhibiting cholinesterase-mediated acetylcholine degradation, and β-sitosterol is a major component in promoting synaptic activity, and it has been shown that β-sitosterol can enhance NGF (nerve growth factor, NGF) activity [45,46] and also reduces Aβ deposition in amyloid APP/PS1 transgenic mice, thereby reducing symptoms of learning memory impairment [47,48]. Ganoderic Acid C can be used to prevent treatment of cognitive impairment in mice and alleviate asthma and lung injury in mice, among others [49,50]. Therefore, all of these 10 active ingredients could be potential active ingredients for the alleviation of neurological-related diseases, and β-sitosterol and ganoderic acid C could be potential active ingredients for the treatment of LMI by GL.

The results of GO functional annotation showed that the regulation of synaptic vesicle exocytosis was the most significant in BP, the gene expression was mostly concentrated on the integral component of presynaptic membrane in CC, and the role of regulating neurotransmitter receptor activity was the most significant in MF, indicating that the improvement of LMI by GL may be because GL first acts on synaptic vesicles, then the neurotransmitters are transferred from the synaptic vesicles through the presynaptic membrane, and finally the activity of neurotransmitters can be regulated.

The KEGG enrichment analysis showed that the 59 action targets of GL to improve LMI were mainly enriched in 55 related pathways. According to the significance of *P*-value, the top 20 pathways with the most significant *P*-value were selected for research. Among these twenty pathways, nine pathways (non-alcoholic fatty liver disease, tuberculosis, rapid and atherosclerosis, pathways in cancer, AGE-RAGE signaling pathway in diabetic complications, hepatitis B, legionellosis, influenza A and human cytomegalovirus infection) are related to various diseases of human beings, three pathways (calcium signaling pathway, IL-17 signaling pathway and taste transduction) are related to signal transduction, two pathways (apoptosis-multiple species and gap junction) are related to cell biological processes, and six pathways (neuroactive ligand–receptor interaction, serotonergic synapse, pathways of neurodegeneration-multiple diseases, Alzheimer’s disease, morphine addiction and dopaminergic synapse) are related to nerves. Furthermore, the document review and the comprehensive analysis indicate that Alzheimer’s disease pathway is the most relevant pathway among the six neuro-related pathways to LMI [51], Therefore, Alzheimer’s disease pathway was selected for study.

In the study of brain biochemical indexes, we found that the content of MDA in the brain serum of mice with learning and memory impairment was significantly increased (*P*<0.01), and the increase of MDA was a clear sign of the onset of ferroptosis [52]. After administration, compared with the MOD group, the MDA content in the serum of the POS, G-H and G-L groups was significantly decreased (*P*<0.01, *P*<0.05). Therefore, GL may improve LMI by inhibiting ferroptosis in neurons.

A PPI network of 59 action targets in GL to improve LMI was constructed first, and then 8 key genes (Tnf, Cnr1, Casp3, Il1b, Nr3c1, Pparg, Chrna7 and Slc6a2) were screened out by using CytoNca and CytoHubb plug-ins in Cytoscape software. It has been shown that Tnf gene is also in the Alzheimer’s disease pathway and plays a major regulatory role. Tnf gene encodes a multifunctional pro-inflammatory cytokines belonging to the TNF superfamily, which plays an important role in the innate immune response and regulation of homeostasis, but it is also related to chronic inflammatory diseases [53]. Some studies have shown that the reduction of Tnf in the hippocampus helps to reduce the inflammatory response and thus improve the learning and memory ability of rats [54]. Therefore, it is believed that Tnf gene may be the most critical factor in the improvement of LMI by GL.

## Conclusion

In summary, two active ingredients (β-sitosterol and ganoderic acid C) directly related to the improvement of LMI of GL were obtained by screening the active ingredients of GL, and triterterpenes of GL may be the key active ingredients for the improvement of LMI. Current studies have shown that GL may act through active ingredients (ergotamine, fumaric acid, lucidumol A, lucialdehyde B, methyl lucidenate F and methyl ganoderate) related to inflammation and immune regulation to reduce Tnf in the hippocampus, reduce inflammation, and improve learning and memory impairment.

## Data Availability

The datasets used and analyzed in the present study are available from the corresponding author.

## Abbreviations

ACH: acetylcholine
DAVID: Database for Annotation Visualization and Integrated Discovery
GL: *Ganoderma lucidum*
GO: Gene Ontology
KEGG: Kyoto Encyclopedia of Genes and Genomes
LMI: learning and memory impairment
MDA: malondialdehyde
PPI: protein–protein interaction
SOD: superoxide dismutase
TNF: tumor necrosis factor
